# Immunosuppressive effects of circulating bile acids in human endotoxemia and septic shock: patients with liver failure are at risk

**DOI:** 10.1186/s13054-023-04620-5

**Published:** 2023-09-27

**Authors:** Julia Leonhardt, Mirrin J. Dorresteijn, Sophie Neugebauer, Diana Mihaylov, Julia Kunze, Ignacio Rubio, Frank-Stephan Hohberger, Silke Leonhardt, Michael Kiehntopf, Klaus Stahl, Christian Bode, Sascha David, Frank A. D. T. G. Wagener, Peter Pickkers, Michael Bauer

**Affiliations:** 1 Department of Anesthesiology and Intensive Care Medicine, Jena University Hospital, Member of the Leibniz Center for Photonics in Infection Research (LPI), Jena, Germany; 2 Center for Sepsis Control and Care (CSCC), Jena University Hospital-Friedrich Schiller University, Jena, Germany; 3 Department of Intensive Care Medicine, Radboud University Medical Center, Nijmegen, the Netherlands; 4 Department of Intensive Care Medicine, Alrijne Hospital, Leiderdorp, the Netherlands; 5 Institute of Clinical Chemistry and Laboratory Diagnostics and Integrated Biobank Jena, Jena University Hospital, Member of the Leibniz Center for Photonics in Infection Research (LPI), Jena, Germany; 6 Department of Oral and Maxillofacial Surgery and Plastic Surgery, Jena University Hospital, Jena, Germany; 7 Department of Hepatology and Gastroenterology, Charité-Universitätsmedizin Berlin, Corporate Member of Freie Universität Berlin, Humboldt-Universität Zu Berlin, Campus Virchow Klinikum, Berlin, Germany; 8 Department of Gastroenterology, Hepatology and Endocrinology, Hannover Medical School, Hannover, Germany; 9 Department of Anesthesiology and Intensive Care Medicine, University Hospital Bonn, Bonn, Germany; 10 Institute of Intensive Care Medicine, University Hospital Zurich, Zurich, Switzerland; 11 Department of Nephrology, Hannover Medical School, Hannover, Germany; 12 Department of Dentistry-Orthodontics and Craniofacial Biology, Research Institute for Medical Innovation, Radboud University Medical Center, Nijmegen, The Netherlands

**Keywords:** Cholestasis, TGR5, GPBAR1, Lipopolysaccharide, LPS, Monocytes, Endotoxin tolerance, Endotoxemia, Immunosuppression

## Abstract

**Background:**

Sepsis-induced immunosuppression is a frequent cause of opportunistic infections and death in critically ill patients. A better understanding of the underlying mechanisms is needed to develop targeted therapies. Circulating bile acids with immunosuppressive effects were recently identified in critically ill patients. These bile acids activate the monocyte G-protein coupled receptor TGR5, thereby inducing profound innate immune dysfunction. Whether these mechanisms contribute to immunosuppression and disease severity in sepsis is unknown. The aim of this study was to determine if immunosuppressive bile acids are present in endotoxemia and septic shock and, if so, which patients are particularly at risk.

**Methods:**

To induce experimental endotoxemia in humans, ten healthy volunteers received 2 ng/kg *E. coli* lipopolysaccharide (LPS). Circulating bile acids were profiled before and after LPS administration. Furthermore, 48 patients with early (shock onset within < 24 h) and severe septic shock (norepinephrine dose > 0.4 μg/kg/min) and 48 healthy age- and sex-matched controls were analyzed for circulating bile acids. To screen for immunosuppressive effects of circulating bile acids, the capability to induce TGR5 activation was computed for each individual bile acid profile by a recently published formula.

**Results:**

Although experimental endotoxemia as well as septic shock led to significant increases in total bile acids compared to controls, this increase was mild in most cases. By contrast, there was a marked and significant increase in circulating bile acids in septic shock patients with severe liver failure compared to healthy controls (61.8 µmol/L vs. 2.8 µmol/L, *p* = 0.0016). Circulating bile acids in these patients were capable to induce immunosuppression, as indicated by a significant increase in TGR5 activation by circulating bile acids (20.4% in severe liver failure vs. 2.8% in healthy controls, *p* = 0.0139).

**Conclusions:**

Circulating bile acids capable of inducing immunosuppression are present in septic shock patients with severe liver failure. Future studies should examine whether modulation of bile acid metabolism can improve the clinical course and outcome of sepsis in these patients.

**Graphical abstract:**

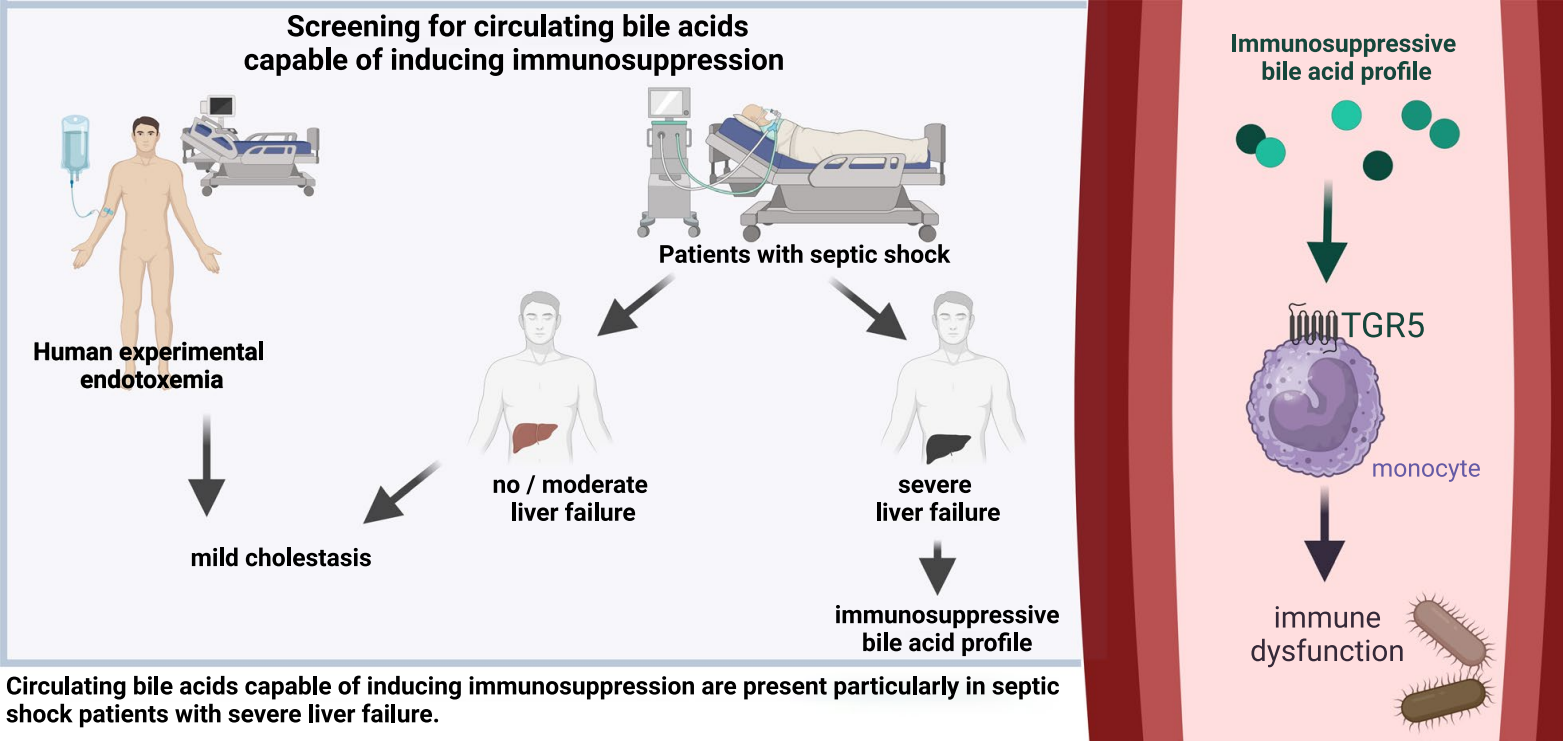

## Background

For many years, bile acids secreted by the liver were thought to function only as emulsifiers of dietary fats. However, the discovery of bile acid receptors has revolutionized our understanding of the physiology of bile acids [1, 2]. Increasing evidence suggests that bile acids also function as signaling molecules or can even be understood as steroid hormones. Bile acid receptors such as the Farnesoid X receptor (FXR) and the G protein-coupled bile acid receptor 1 (GPBAR1, also known as TGR5) regulate metabolism and immunity, respectively [1–4].

The immunomodulatory TGR5 is expressed by monocytes and macrophages [1, 5]. These innate immune cells lose their ability to phagocytose and produce pro-inflammatory cytokines when TGR5 is activated [1, 6, 7]. Apart from monocytes, other leukocytes show little to no expression of bile acid receptors [5, 8]. Activation of TGR5 in monocytes is dependent on the exact bile acid composition and quantity. Generally speaking, secondary bile acids are more potent TGR5 agonists than primary bile acids [1, 9]. For example, taurolithocholic acid (a secondary bile acid) is 20- to 200 times more potent than cholic acid (a primary bile acid) [1, 7].

Physiological bile acid compositions that activate TGR5 are found in the intestine but not in the blood. In cholestasis, however, the bile acid flow is disrupted leading to the retention and spill-over of bile acids into the blood. A recent study by us showed that increased circulating bile acids in liver failure patients activate TGR5, leading to significant immune dysfunction. The study detected circulating immunosuppressive bile acids in patients with acute-on-chronic liver failure, acute liver failure and liver graft failure [7]. However, increased circulating bile acids are frequently detected in critically ill patients, particularly in those with septic shock [10, 11]. Whether the bile acid compositions that appear in patients with septic shock can activate TGR5 and thus contribute to sepsis-induced immunosuppression has yet to be evaluated.

In animal models, endotoxins (such as bacterial lipopolysaccharide) and the subsequent release of inflammatory cytokines (particularly IL-6 and TNFα) lead to the disruption of hepatocellular bile acid excretion and increased circulating bile acids [12–14]. Likewise, it is assumed that endotoxemia, and subsequent cytokine storm, are the drivers of increased circulating bile acids in human sepsis.

This study was therefore designed to determine whether the composition of circulating bile acids in human endotoxemia and septic shock activates the immunosuppressive receptor TGR5 and, if so, which patients are at risk.

## Patients and methods

### Sepsis patients and healthy controls

To study the effect of endotoxemia on bile acid profiles, ten healthy male non-smoking volunteers were recruited and experimental human endotoxemia was conducted. Briefly, subjects were admitted to the Research Intensive Care Unit of the Radboud University Medical Centre, Nijmegen, the Netherlands. Cannulation of the brachial artery and the antecubital vein was performed for invasive blood pressure monitoring and intravenous fluid or drug administration, respectively. After fluid loading (1.5 L crystalloid intravenously), continuous infusion of 150 mL/h crystalloid was initiated with continuous monitoring of vital signs, including body temperature, which was measured using an infrared tympanic thermometer. Purified lipopolysaccharide (LPS, US Standard Reference Endotoxin *E. coli* O:113, obtained from the Pharmaceutical Development Section of the National Institutes of Health (Bethesda, MD, USA)) was administered at a dose of 2 ng/kg body weight. At the indicated times, peripheral blood samples were collected for analysis of bile acid profiles, cytokines, C-reactive protein (CRP) and total bilirubin. Circulating cytokines were measured using a multiplex assay according to the manufacturer’s instructions (Bio-Plex, Bio-Rad Laboratories, Hercules, CA, USA).

To study the effect of bile acid profiles in septic shock, serum samples from 48 patients enrolled in two related clinical trials investigating the therapeutic effect of total plasma exchange were collected [15, 16]. To exclude treatment effects, all blood samples for our study were taken prior to total plasma exchange. All laboratory (including total serum bilirubin) and clinical data (including scores) were obtained at the time of blood collection and before plasma exchange. Inclusion criteria for patients with septic shock were: (1) age ≥ 18 years, (2) sepsis according to the SEPSIS-3 definition [17], (3) profound systemic hypotension requiring ≥ 0.4 µg/kg/min norepinephrine despite adequate intravenous fluid resuscitation of at least 30 mL crystalloids per kg bodyweight, and iv) onset of vasopressor use < 24 h prior to screening. Exclusion criteria were pregnancy and breast feeding. Liver failure in patients with septic shock was assessed according to the individual hepatic Sequential Organ Failure Assessment (SOFA) sub-score using serum bilirubin. A hepatic SOFA score of ≥ 3 was defined as liver failure as described before [18, 19].

Furthermore, serum samples from 48 age- and sex-matched healthy volunteers with no signs of infection within the last 14 days and no past medical history of cholestasis or liver diseases were used as controls.

The study and transfer of samples between study centers were approved by the local ethical committees of all study centers (Hannover Medical School: No. 2786-2015 and No. 8852_MPG_23b_2020, University Hospital Bonn: No. 024/20, Radboud University Medical Center: 2009/047 and NL27052.091.09, University Hospital Jena: 2022-2571-Material and 2022-2606-Material). Informed consent was obtained from all volunteers and patients or their legal representatives before inclusion in the study.

### Bile acids

Individual bile acid profiles were assessed using an LC–MS/MS in-house assay. Bile acid standards were purchased from VWR International GmbH (Darmstadt, Germany), TCI Deutschland GmbH (Eschborn, Germany) and Sigma-Aldrich Chemie GmbH (Taufkirchen, Germany) and had a purity of at least of 91%. HPLC-grade methanol, ethanol, ammonium acetate and formic acid were obtained from Carl Roth (Karlsruhe, Germany), Merck KGaA (Darmstadt, Germany) and Sigma-Aldrich Chemie GmbH (Taufkirchen, Germany). 270 µL of 85% aqueous methanol was added to 30 µL sample in a Thomson Single Step® Filter Vial (PES membrane 0.2 µM, Thomson Instrument Company, California). This solution was mixed for 20 s, centrifuged at 200×*g* for 1 min, filtered and placed in the autosampler. An Agilent 1200 high performance liquid chromatography system (Agilent Technologies GmbH, Germany) with a CTC-PAL autosampler coupled to an API 4000 Triple Quadrupole mass spectrometer with electrospray ionization source (AB Sciex, Germany) was used for quantification. All chromatographic separations were performed with a reverse-phase Agilent Zorbax Eclipse XDB-C18 (3.5 µm, 100 × 3 mm) analytical column equipped with a guard column (C18, 4 × 3 mm; Phenomenex, Aschaffenburg, Germany). The mobile phase consisted of water (A) and methanol (B), both containing 0.012% formic acid and 5 mM ammonium acetate, at a total flow rate of 300 µL/min. Total bile acids were calculated as the sum of individual bile acids.

### Statistical analysis

Statistical analysis was performed using GraphPad Prism 9.4.1 (La Jolla, CA, USA) or SPSS (IBM, Chicago, IL, USA). Continuous variables are represented as median and 25–75% interquartile range (IQR). The Wilcoxon signed rank test (nonparametric) or two-tailed, paired Student’s t-test (parametric data) was used for comparisons between two matched groups. Kruskal–Wallis one-way analysis of variance on ranks with Dunn’s post-hoc test was used to compare the effects of multiple groups. Comparisons between multiple time points were made with one-way repeated measures analysis of variance with Geisser-Greenhouse correction and Dunnett’s post-hoc test. Correlations were calculated using Spearman coefficients. For all comparisons, *p* < 0.05 was considered significant. The graphical abstract and figures were created with BioRender.com.

## Results

### Experimental endotoxemia induces sepsis-like symptoms and an increase in markers of inflammation and cholestatic liver dysfunction

**Fig. 1:**
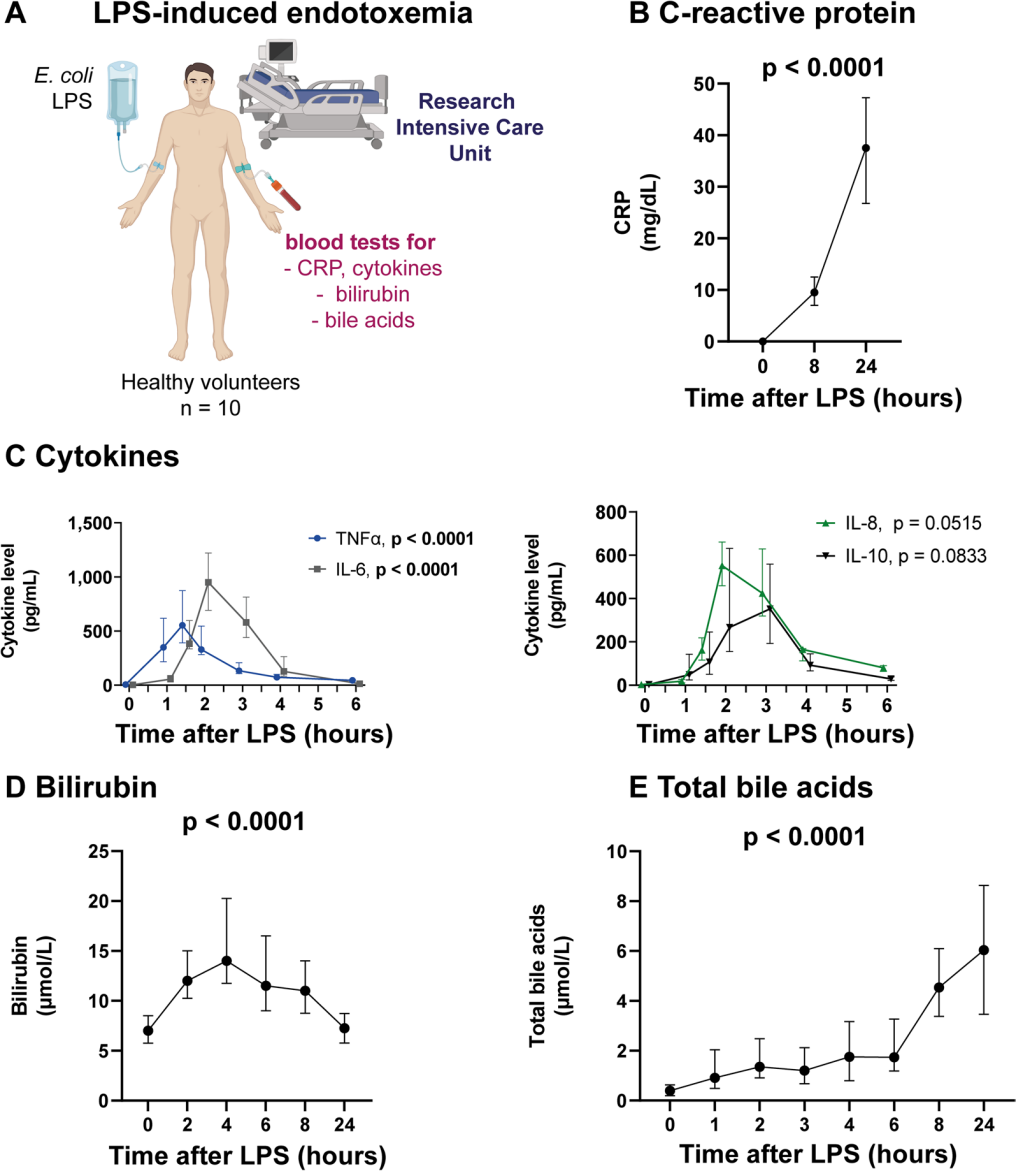
Experimental endotoxemia induces sepsis-like symptoms and an increase in markers of inflammation and cholestatic liver dysfunction. **A**–**E** Ten healthy volunteers received 2 ng *E. coli* LPS per kg body weight. **A** The figure was created with BioRender.com. **B**–**E** C-reactive protein, cytokines, bilirubin and total bile acids were assessed. Graphs show median and IQR. One-way repeated measures analysis of variance. *CRP* C-reactive protein, *LPS* lipopolysaccharide

To study the effects of endotoxemia on bile acids, ten healthy volunteers were admitted to our research intensive care unit and received *E. coli* lipopolysaccharide (LPS) intravenously (Fig. 1A). Baseline characteristics (age and sex) of healthy volunteers can be found in Table 1. Administration of intravenous LPS resulted in sepsis-like symptoms (e.g., shivering and muscle pain) in all subjects. Furthermore, we observed a significant change in systemic hemodynamics (Table 2), C-reactive protein (CRP, Fig. 1B) and circulating cytokines (Fig. 1C).

**Table 1:** Characteristics of healthy volunteers with LPS-induced endotoxemia, patients with septic shock and age- and sex-matched healthy controls

	Healthy volunteers with LPS-induced endotoxemia	Septic shock	Healthy controls	Septic shock versus healthy controls (*p* value)
*n* =	10	48	48	
Male (*n*, %)	10 (100)	30 (63)	30 (63)	1.0
Age (years)	22.5 (21.5–25.3)	53.5 (47.0–61.5)	53.5 (45.0–62.5)	1.0
*Site of infection (n, %)*				
Pneumonia		26 (54)		
Endocarditis		2 (4)		
Soft tissue		4 (8)		
Abdomen		14 (29)		
Other sepsis		2 (4)		
*Identified pathogen (n, %)*				
Gram-positive bacteria		10 (21)		
Gram-negative bacteria		15 (31)		
Fungal		5 (10)		
Viral		3 (6)		
Mixed		6 (13)		
Non-identified		9 (19)		
SOFA score		17 (14–19)		
NE dose (µg/kg/min)		0.66 (0.46–0.93)		
Lactate (mmol/L)		4.0 (2.7–7.0)		
Bilirubin (µmol/L)		29.0 (13.5–51.8)		
CRP (mg/L)		248 (131–343)		
PCT (µg/L)		19.7 (6.4–53.4)		
WBC (10^3^/µL)		13.5 (1.7–21.4)		

Continuous variables are expressed as median and IQR

*CRP* C-reactive protein, *LPS* lipopolysaccharide, *NE* norepinephrine, *PCT* procalcitonin, *SOFA* Sequential Organ Failure Assessment, *WBC* white blood cell count. Wilcoxon signed-rank test

**Table 2:** Changes in vital signs in healthy volunteers receiving LPS

	Before LPS	4 h after LPS	*p* value
Heart rate (bpm)	69 (63–83)	92 (83–102)	**0.0006**
MAP (mmHg)	89 (87–101)	77 (74–83)	**0.0002**
Temperature (°C)	37.0 (36.5–37.2)	37.9 (37.8–38.5)	**0.0018**

Significant *p* values are in bold

Data are expressed as median and IQR

*Bpm* beats per minute, *MAP* mean arterial pressure

Two-tailed, paired Student’s *t* test

In sepsis and critical illness, circulating bilirubin and bile acids are well established markers of cholestatic liver dysfunction [20, 21]. Healthy volunteers developed a mild increase in these markers after onset of experimental endotoxemia. Bilirubin levels peaked after 4 h (Fig. 1D, 14.0 µmol/L vs. 7.0 µmol/L at baseline, *p* = 0.0003). Total bile acids rose steadily over time (Fig. 1E). The most significant increase in total bile acid levels was found 8 h after LPS infusion (4.5 µmol/L vs. 0.4 µmol/L before LPS infusion).

### Experimental endotoxemia induces changes in bile acid profiles

**Fig. 2:**
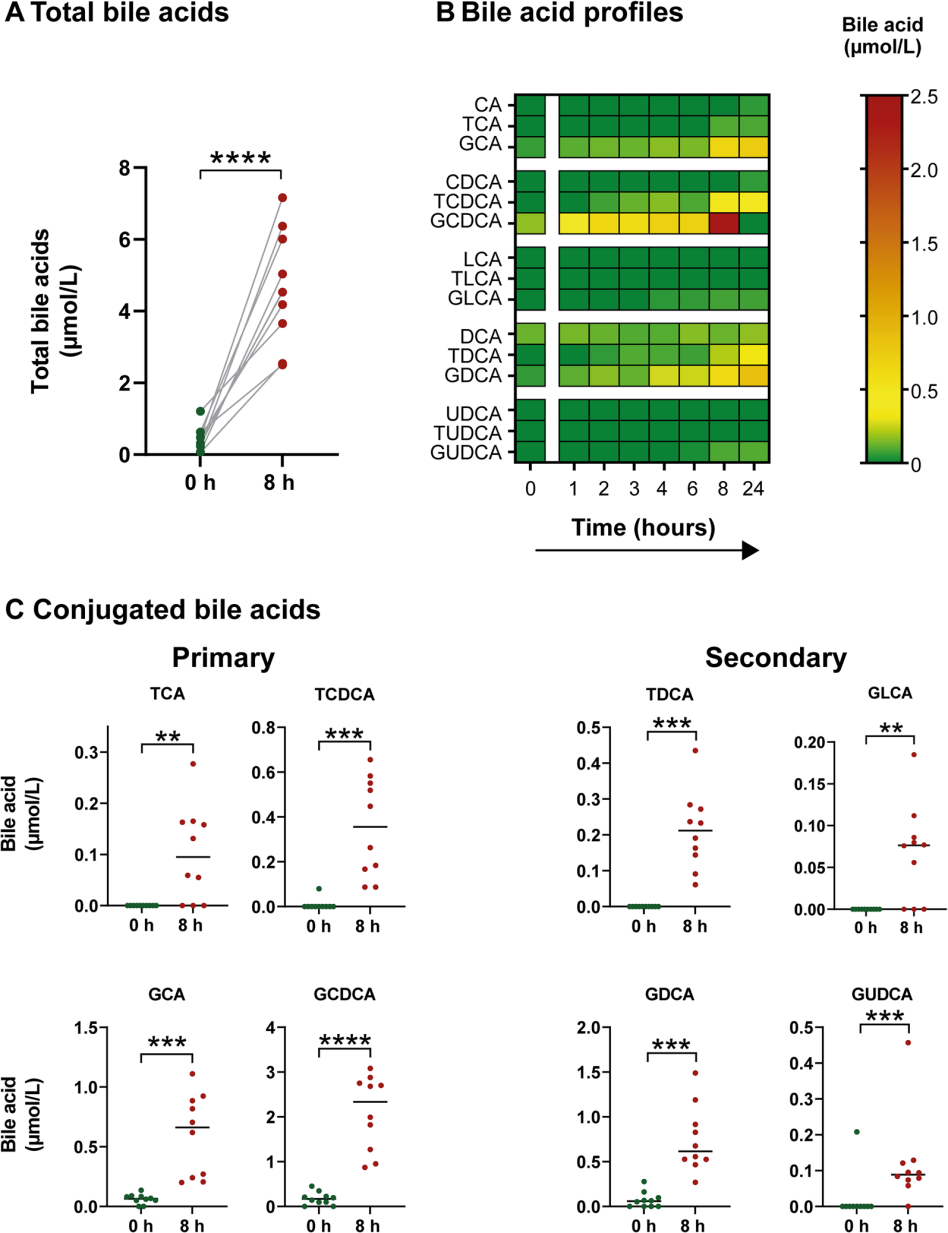
Experimental endotoxemia induces changes in bile acid profiles. **A**–**C** Total bile acids and bile acid profiles of ten healthy volunteers were assessed before and after IV administration of 2 ng *E. coli* LPS per kg body weight. **A** Total bile acids before and 8 h after LPS infusion. **B** Heat map of all measured bile acids at different time points. **C** Graphs of all bile acids with significant differences before and 8 h after LPS infusion. Each dot represents the value for a single subject at a given time point. The horizontal line represents the median. Two-tailed, paired Student’s *t* test. **p < .01, ***p < .001, ****p < .0001. *CA* cholic acid; *CDCA* chenodeoxycholic acid; *DCA* deoxycholic acid; *GCA* glycocholic acid; *GCDCA* glycochenodeoxycholic acid; *GDCA* glycodeoxycholic acid; *GLCA* glycolithocholic acid; *GUDCA* glycoursodeoxycholic acid; *LCA* lithocholic acid; *TCA* taurocholic acid; *TCDCA* taurochenodeoxycholic acid; *TDCA* taurodeoxycholic acid; *TLCA* taurolithocholic acid; *TUDCA* tauroursodeoxycholic acid; *UDCA* ursodeoxycholic acid

We further analyzed the increase in total bile acids by assessing the individual bile acid profiles of our healthy volunteers with experimental endotoxemia. While unconjugated bile acids remained unchanged, most conjugated bile acids were significantly increased (Fig. 2B, C). Conjugated primary bile acids (i.e., GCA, TCA, GCDCA and TCDCA) were the predominant circulating bile acids, accounting for 71% of total bile acids (3.2 µmol/L of 4.5 µmol/L total bile acids 8 h after LPS infusion, Fig. 2C).

### Experimental endotoxemia increases immunosuppressive bile acids

**Fig. 3:**
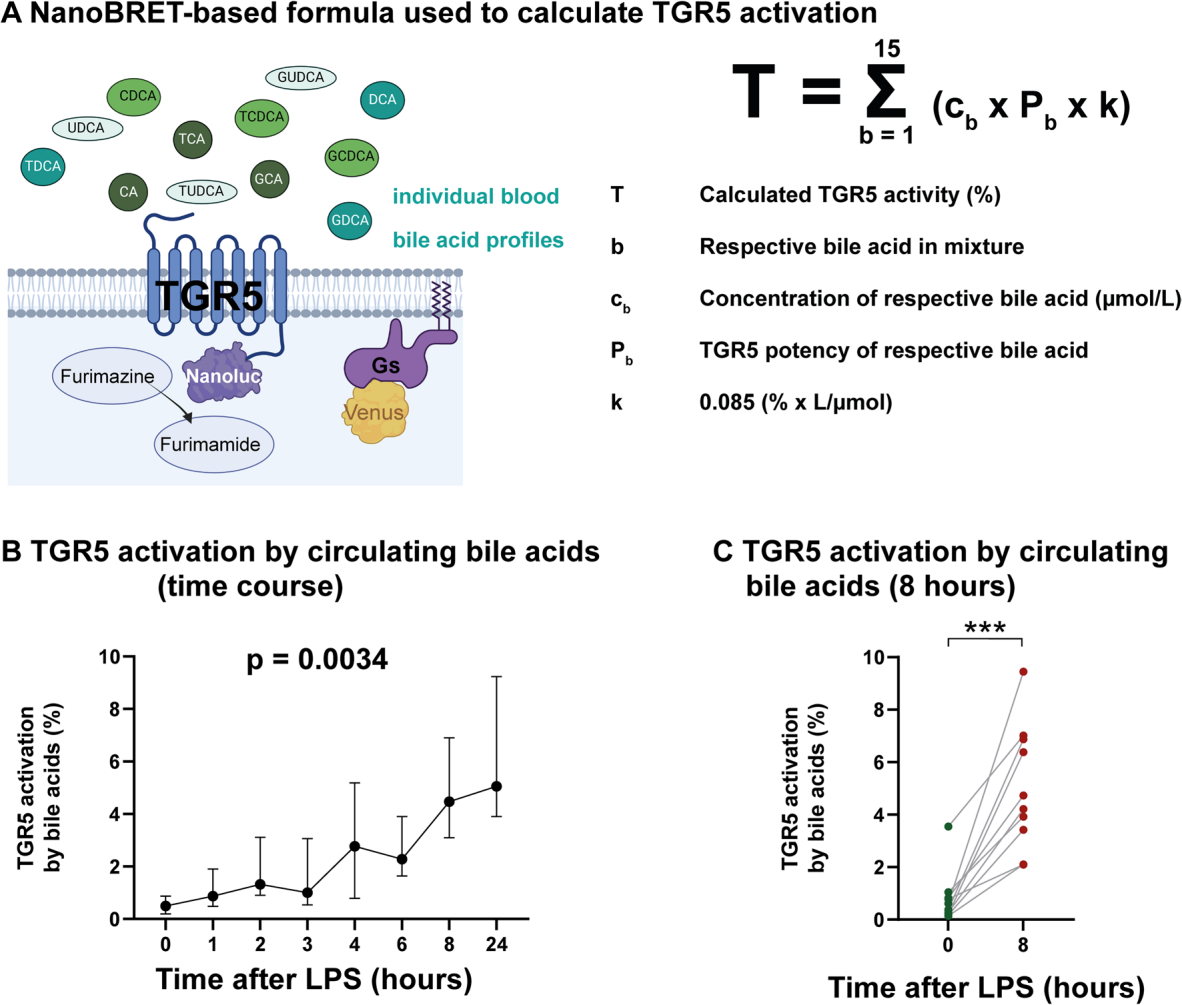
Experimental endotoxemia increases immunosuppressive bile acids. **A**–**C** Bile acid profiles of ten healthy volunteers were assessed before and after IV administration of 2 ng *E. coli* LPS per kg body weight. TGR5 activation by the individual bile acid profile was calculated as described previously (7). **A** NanoBRET-based formula used to calculate TGR5 activity. The figure was created with BioRender.com. **B** TGR5 activation induced by circulating bile acids after LPS infusion. Graph shows mean and IQR. One-way repeated measures analysis of variance. **C** TGR5 activation induced by circulating bile acids 8 h after LPS administration. Each dot represents the value for a single subject at a given time point. Two-tailed, paired Student’s *t* test. ***p < .001. *LPS* lipopolysaccharide

To investigate, whether the complex compositions of circulating bile acids found in experimental endotoxemia could indeed activate TGR5, we calculated TGR5 activity using the NanoBRET-based formula described previously (Fig. 3A) [7]. The formula takes into account the concentration of each individual bile acid and its respective potency to activate TGR5. A bile acid profile significantly activating TGR5 was found in experimental endotoxemia (Fig. 3B). However, in line with the weak increases in bilirubin and total bile acids that we observed, TGR5 activation was mild: 4.5% 8 h after vs. 0.5% before LPS infusion (*p* = 0.0003).

### Circulating bile acids are significantly increased in septic shock

**Fig. 4:**
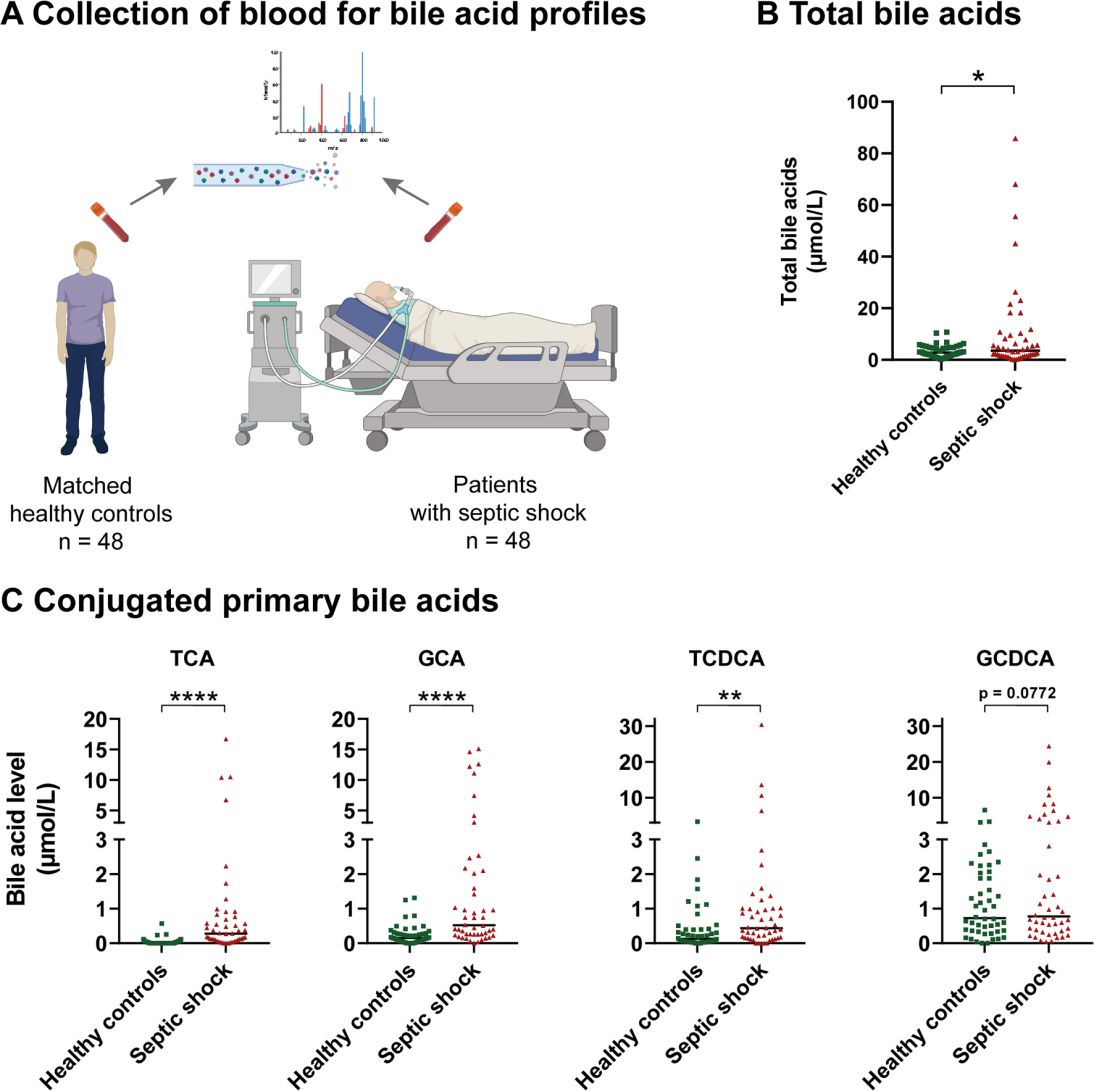
Bile acid profiles of healthy controls and patients with septic shock. **A**, Bile acid profiles of 48 patients with septic shock and 48 age- and sex-matched controls were measured by mass spectrometry. The figure was created with BioRender.com. **B**, Total bile acids were significantly increased in patients with septic shock. **C**, The increase of total bile acids was due to a significant increase in primary conjugated bile acids. Each dot represents the value for a single subject at a given time point. The horizontal line represents the median. Wilcoxon signed-rank test. *p < .05, **p < .01, ****p < .0001. *GCA* glycocholic acid, *GCDCA* glycochenodeoxycholic acid, *TCA* taurocholic acid, *TCDCA* taurochenodeoxycholic acid

We further assessed whether the effects observed in experimental endotoxemia would also be detectable in critically ill patients with sepsis. Septic patients admitted to our intensive care units were screened for early (shock onset within < 24 h) and severe septic shock (norepinephrine dose > 0.4 μg/kg/min). Forty-eight patients were identified, and a group of 48 age- and sex-matched controls was selected accordingly (Fig. 4A, Table 1).

Patients with septic shock showed significant differences in bile acid quantity and composition compared to healthy controls (Table 3 and Fig. 4). Their total bile acid level was significantly higher than that of healthy controls (Fig. 4B, 3.51 µmol/L vs. 2.78 µmol/L, *p* = 0.0139). This increase was due to significantly elevated conjugated primary bile acids (e.g., taurocholic acid, TCA and glycocholic acid, GCA, Fig. 4C). By contrast, secondary bile acids, which were present in low quantities in healthy controls, were even lower in patients with septic shock (Table 3).

**Table 3:** Detailed bile acid profiles of healthy controls and patients with septic shock

	Septic shock	Healthy controls	Septic shock versus healthy controls (*p*-value)
Total bile acids (µmol/L)	3.51 (1.56–10.14)	2.78 (1.38–4.81)	**0.0139**
*Primary bile acids (µmol/L)*			
CA	0.02 (0.00–0.05)	0.03 (0.01–0.11)	0.5535
TCA	0:28 (0.07–0.75)	0.00 (0.00–0.04)	** < 0.0001**
GCA	0.52 (0.26–2.08)	0.16 (0.06–0.29)	** < 0.0001**
CDCA	0.01 (0.00–0.11)	0.11 (0.00–0.24)	0.3412
TCDCA	0.43 (0.15–1.01)	0.13 (0.04–0.40)	**0.0067**
GCDCA	0.78 (0.36–3.32)	0.73 (0.34–1.99)	0.0772
*Secondary bile acids (µmol/L)*			
LCA	0.00 (0.00–0.00)	0.03 (0.00–0.04)	** < 0.0001**
TLCA	0.00 (0.00–0.00)	0.00 (0.00–0.00)	0.0625
GLCA	0.00 (0.00–0.00)	0.01 (0.00–0.03)	** < 0.0001**
DCA	0.00 (0.00–0.40)	0.20 (0.04–0.37)	** < 0.0001**
TDCA	0.00 (0.00–0.09)	0.06 (0.00–0.16)	0.3721
GDCA	0.06 (0.00–0.22)	0.22 (0.09–0.53)	**0.0008**
UDCA	0.00 (0.00–0.04)	0.04 (0.02–0.13)	**0.0103**
TUDCA	0.00 (0.00–0.01)	0.00 (0.00–0.00)	**0.0484**
GUDCA	0.07 (0.02–0.36)	0.06 (0.01–0.14)	**0.0486**

Significant *p* values are in bold

Data are expressed as median and IQR

*CA* cholic acid, *CDCA* chenodeoxycholic acid, *DCA* deoxycholic acid, *GCA* glycocholic acid, *GCDCA* glycochenodeoxycholic acid, *GDCA* glycodeoxycholic acid, *GLCA* glycolithocholic acid, *GUDCA* glycoursodeoxycholic acid, *LCA* lithocholic acid, *TCA* taurocholic acid, *TCDCA* taurochenodeoxycholic acid, *TDCA* taurodeoxycholic acid, *TLCA* taurolithocholic acid, *TUDCA* tauroursodeoxycholic acid, *UDCA* ursodeoxycholic acid. Wilcoxon signed-rank test

### Patients with severe liver failure show a marked increase in circulating bile acids

**Fig. 5:**
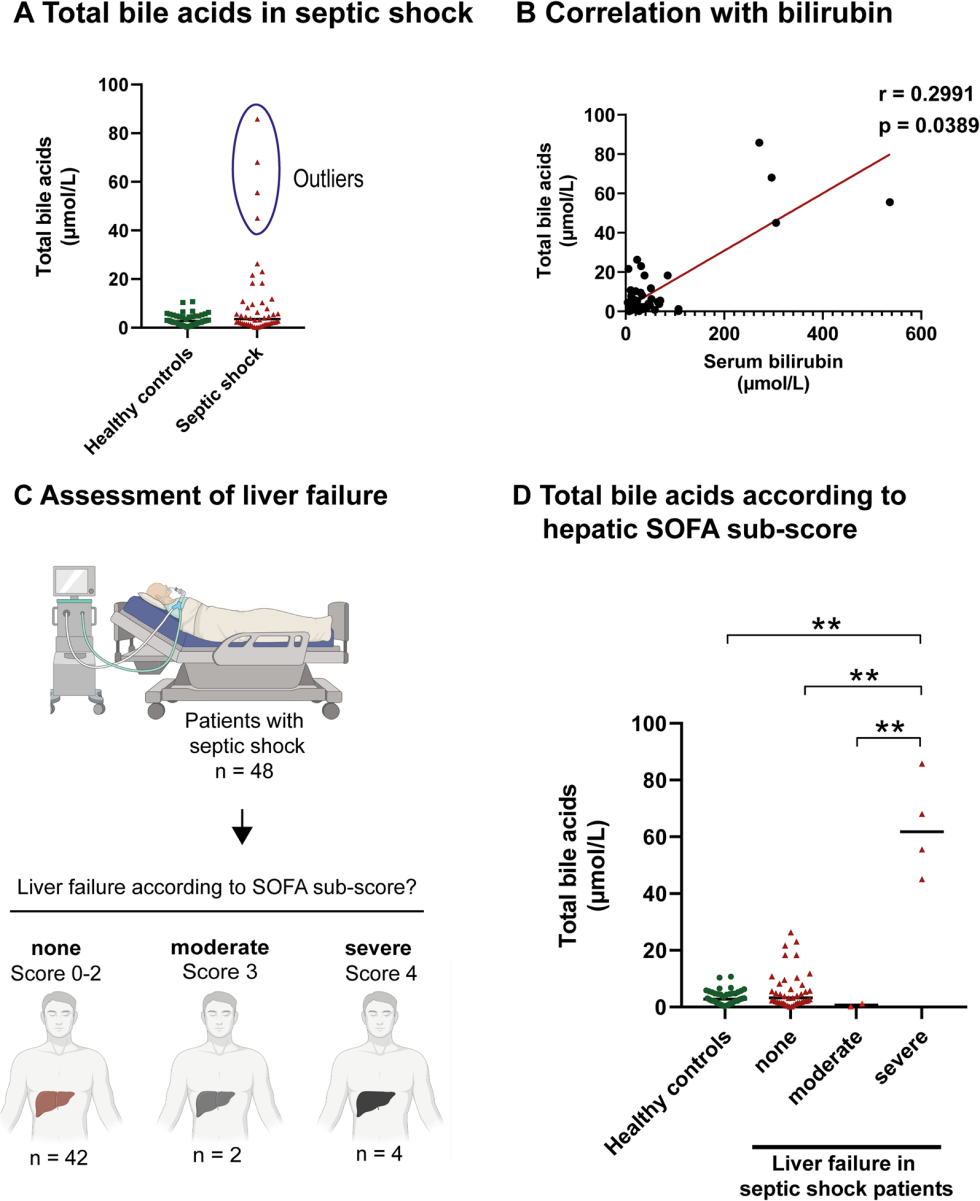
Total bile acids in septic patients with severe liver failure. **A** Total bile acids in 48 patients with septic shock versus 48 age- and sex-matched controls. Grubbs test identified four outliers in the septic shock group. Each dot represents the value of a single subject, the horizontal line represents the median. **B** Total bile acid levels correlated significantly with serum bilirubin. Values for Spearman’s rank correlation coefficient (*r*) are given. **C** Patients with septic shock were grouped according to the presence and severity of liver failure, as indicated by the hepatic SOFA sub-score. The figure was created with BioRender.com. **D** Total bile acids in patients with and without liver failure. Each dot represents the value of a single subject, the horizontal line represents the median, Kruskal–Wallis one-way analysis of variance. **p < .01. *SOFA* Sequential Organ Failure Assessment

Most patients with septic shock had shown a mild increase in circulating bile acids similar to that of human subjects with experimental endotoxemia. However, the patient population was heterogeneous. Grubbs test detected four outliers with markedly increased circulating bile acids (Fig. 5A). In septic shock patients, increased levels of circulating bile acids may not only reflect cholestatic liver dysfunction due to endotoxemia and cytokine storm, but also liver failure. Bilirubin is the best-established laboratory parameter for the detection and scoring of liver failure in septic patients [20, 22]. In line with this, total bile acids closely correlated with bilirubin levels in our patients (Fig. 5B). The Sequential Organ Failure Assessment (SOFA) score uses serum bilirubin levels to indicate and discriminate liver failure in sepsis. We therefore grouped our patients according to the presence and severity of liver failure using the hepatic SOFA sub-score (Fig. 5C). Four patients were classified as having severe liver failure. Strikingly, these four patients were identical with the outliers in Fig. 5A. In line with this, patients with severe liver failure showed significantly increased circulating bile acids (Fig. 5D).

The patients’ past medical history was also examined. All patients with severe liver failure had advanced preexisting liver diseases before developing septic shock: two had liver cirrhosis, one had hepatic graft-versus-host disease (GvHD), and one had acute alcoholic steatohepatitis. By contrast, none of the patients with moderate liver failure and only 6 of the 42 patients (14.3%) without liver failure had a past medical history of liver disease, including liver cirrhosis (*n* = 4), autoimmune hepatitis (*n* = 1), and secondary sclerosing cholangitis (*n* = 1).

### Patients with severe liver failure have circulating immunosuppressive bile acids

**Fig. 6:**
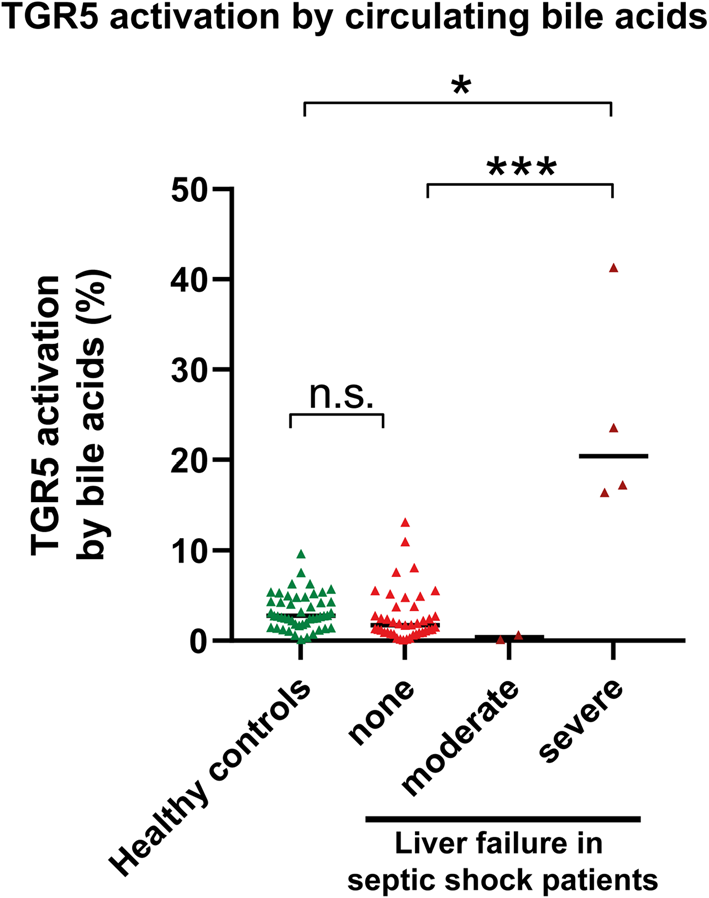
Patients with severe liver failure have TGR5-activating bile acid profiles. Patients with severe liver failure showed significant extrapolated TGR5 activation by circulating bile acids. Each dot represents the value of a single subject. The horizontal line represents the median, Kruskal–Wallis one-way analysis of variance. *n.s.* not significant, *p < .05, ***p < .001

Although the quantity of total bile acids in septic patients generally was significantly increased, the levels of highly immunosuppressive secondary bile acids were decreased (Table 3). Consequently, the capability of circulating bile acids to induce TGR5 activation was comparable between septic shock patients without liver failure and healthy controls (Fig. 6). By contrast, patients with severe liver failure showed a bile acid profile inducing significant TGR5 activation: 20.4% vs. 1.7% compared to patients without liver failure (*p* = 0.0006) and 20.4% versus 2.8% compared to healthy controls (*p* = 0.0139).

## Discussion

This study shows that septic shock patients develop cholestasis with significantly increased bile acids. Two main causes of cholestasis in septic shock patients were analyzed in this study: endotoxemia and liver failure. Endotoxemia with subsequent cytokine storm and systemic inflammation led to a significant but mild increase in circulating bile acids. A similar pattern was found in most patients with septic shock. By contrast, septic shock patients with liver failure showed markedly increased bile acid levels, which were capable of inducing significant TGR5 activation.

The accumulation of circulating bile acids is an early and critical event in sepsis. In animal models, increased levels of circulating bile acids have been detected as early as 1 h after induction of experimental sepsis [23]. In line with this, our data show a significant increase in circulating bile acids within 8 h after LPS infusion in experimental endotoxemia. A significant and early increase in circulating bile acids (within 24 h of onset of vasopressor use) was also observed in our septic shock patients.

Conjugated primary bile acids (GCA, TCA, GCDCA and TCDCA) were the main drivers of bile acid accumulation in experimental endotoxemia and septic shock patients. These findings are consistent with those of previous studies investigating circulating bile acids in sepsis, acute respiratory distress syndrome (ARDS) or critically ill patients [10, 11, 24, 25]. Endotoxemia and subsequent cytokine storm have been reported to disrupt hepatocellular bile acid export. In particular, a marked downregulation of the canalicular bile salt export pump (BSEP) and of the multidrug resistance-associated protein 2 (MRP2) has been observed [11–14, 26–29]. However, despite the strong disturbance of bile acid export, hepatocytes are still able to synthesize and conjugate bile acids with glycine and taurine [11]. Consequently, conjugated bile acids that are either enterohepatically recirculated or de novo synthesized, accumulate in hepatocytes and spill over into the blood.

Interestingly, our data show that the conjugated secondary bile acids (taurine- and glycine-conjugated DCA, LCA and UDCA) were significantly increased in experimental endotoxemia but not in septic shock. Secondary bile acids originate from primary bile acids that are excreted into the intestine and transformed by the gut microbiota. Reabsorption and recycling by the enterohepatic circulation raise secondary bile acids to detectable levels in the blood. The disruption of canalicular bile acid export by endotoxemia may consequently lead to increased levels of circulating secondary bile acids. However, unlike healthy volunteers, patients with septic shock were treated with antibiotics. Antibiotic treatments have been shown to reduce gut microbiota and, thus, decrease circulating secondary bile acids by up to 1000-fold [30–32]. Therefore, the marked reduction in secondary bile acids caused by antibiotics could have outweighed the effects of endotoxemia in our patients.

Although TGR5-activating circulating bile acids were found in experimental endotoxemia, this effect was mild (4.5% receptor activation), though significant. Strikingly, TGR5-activating circulating bile acids were absent in septic shock patients without liver failure due to the lack of highly immunosuppressive secondary bile acids. By contrast, circulating bile acids in patients with severe liver failure were capable of activating TGR5 significantly and relevantly (20.4% receptor activation). TGR5 activation has been reported to induce monocyte dysfunction, as characterized by a decrease in the release of tumor necrosis factor α (TNFα), IL-1β and IL-6 upon LPS stimulation and an unaltered release of anti-inflammatory IL-10 [1, 7]. This monocyte dysfunction is associated with increased mortality [7].

Although advanced intensive care medicine and early goal-directed therapies have improved survival rates from the primary septic hyper-inflammatory phase, sepsis is still the leading cause of in-hospital death in Western societies [33–35]. The high mortality of sepsis is at least partly due to a secondary anti-inflammatory phase, which is characterized by increased susceptibility to opportunistic infections [36]. This sepsis-induced immunosuppression is typically characterized by dysfunctional monocytes, which show a decreased release of pro-inflammatory cytokines such as tumor necrosis factor α (TNFα), IL-1β and IL-6 upon stimulation with LPS and an enhanced secretion of anti-inflammatory IL-10 [37, 38]. Strikingly, immunosuppressive bile acids induce an identical immune phenotype in monocytes [1, 7]. Several attempts have been made to boost septic patients’ innate immune system with cytokines (e.g., IFN-γ), growth factors (e.g., GM-CSF) or pathogen-associated molecular patterns (β-glucan) [39–42]. However, a specific treatment that prevents septic immunosuppression is still lacking. Therefore, the identification of mechanisms leading to the impairment of monocyte function in sepsis is of paramount importance.

Circulating bile acids closely correlate with mortality in patients with sepsis and critical illness [10, 43, 44]. Indeed, circulating bile acids are better predictors of sepsis-related mortality than liver function parameters, such as bilirubin [10, 43]. Therefore, several authors speculated that bile acids play an active role in the pathogenesis of sepsis [10, 29, 45]. Moreover, studies have shown that HMG-CoA reductase inhibitors (drugs that significantly inhibit cholesterol and, subsequently, bile acid synthesis) reduce mortality in sepsis and infection-related ARDS [46–48]. However, the results of the available studies and meta-analyses of the effects of statins on sepsis are inconsistent [49, 50]. The beneficial effect of statins may at least partially be due to a reduction of circulating immunosuppressive bile acids in a subset of septic patients.

Our study shows for the first time that bile acids capable of inducing immunosuppression are present in a subset of septic patients. Our findings suggest that marked TGR5 activation is restricted to septic patients with liver failure. These patients may benefit from therapeutic approaches reducing circulating bile acids. As mentioned above, HMG-CoA reductase inhibitors such as simvastatin can efficiently reduce bile acid synthesis [51, 52]. Furthermore, the enterohepatic circulation of bile acids can be inhibited by bile acid sequestrants, such as cholestyramine and colesevelam [53–55]. Direct elimination of circulating bile acids can be achieved by albumin dialysis or total plasma exchange [56, 57]. Apart from simply removing bile acids from the circulation, future therapies might directly target the sepsis-induced disruption of the excretory liver function. Accordingly, we recently showed that liver-specific inhibition of phosphatidylinositol-3-kinase (PI3K) restored both the canalicular architecture of hepatocytes and biliary excretion [58]. However, these therapies may only be appropriate as part of an individualized approach for patients with circulating immunosuppressive bile acids rather than as a “one size fits all” concept for septic patients.

Our study has several limitations. First and foremost, the sample size is too small to draw conclusions about the consequences of circulating immunosuppressive bile acids in patients. Therefore, our results on the occurrence of circulating immunosuppressive bile acids in septic shock were intended to be hypothesis-generating and a useful aid for designing a larger ongoing follow-up study. The follow-up study will thus be appropriate for investigating the consequences of circulating immunosuppressive bile acids, such as mortality or secondary infections, and for evaluating potential therapeutic options. Furthermore, the present study used the SOFA score to screen septic shock patients for the presence of liver failure, as described before [18, 19]. This simple and pragmatic score defines liver failure by serum bilirubin [59] and does not distinguish different forms or causes of liver failure. Moreover, it does not discriminate between patients with and without pre-existing liver diseases. However, the SOFA score is the best-established and best-evaluated method of screening for organ failure in septic patients [17, 22]. In our study, it sufficiently identified all patients at risk for circulating immunosuppressive bile acids.

## Conclusions

Septic shock patients with severe liver failure develop cholestasis with a massive increase in circulating bile acids. These circulating bile acids are capable of activating the immunosuppressive bile acid receptor TGR5. Future studies should evaluate the potential of HMG-CoA reductase inhibitors, albumin dialysis and other therapies to reduce immunosuppressive bile acids and their effects on sepsis outcome.

## Data Availability

The datasets used and analyzed are available from the corresponding author on reasonable request.

## Abbreviations

Bpm: Beats per minute
MAP: Mean arterial pressure
BSEP: Bile salt export pump
CA: Cholic acid
CDCA: Chenodeoxycholic acid
CRP: C-reactive protein
DCA: Deoxycholic acid
GCA: Glycocholic acid
GCDCA: Glycochenodeoxycholic acid
GDCA: Glycodeoxycholic acid
GLCA: Glycolithocholic acid
GPBAR1: G protein-coupled bile acid receptor 1 (aka TGR5)
GUDCA: Glycoursodeoxycholic acid
HMG-CoA: 3-Hydroxy-3-methyl-glutaryl-coenzyme A
IL: Interleukin
LCA: Lithocholic acid
LPS: Lipopolysaccharide
MRP2: Multidrug resistance associated protein 2
NanoBRET: Bioluminescence resonance energy transfer using nanoluciferase
NE: Norepinephrine
PCT: Procalcitonin
SOFA: Sequential Organ Failure Assessment
TCA: Taurocholic acid
TCDCA: Taurochenodeoxycholic acid
TDCA: Taurodeoxycholic acid
TGR5: The G protein-coupled bile acid receptor TGR5 (aka GPBAR1)
TLCA: Taurolithocholic acid
TNFα: Tumor necrosis factor α
TUDCA: Tauroursodeoxycholic acid
UDCA: Ursodeoxycholic acid
WBC: White blood cell count
